# 1472. Development of an Interactive Provider Dashboard for Enhanced Recovery After Surgery to Monitor Compliance with Perioperative Surgical Site Infection Measures

**DOI:** 10.1093/ofid/ofad500.1308

**Published:** 2023-11-27

**Authors:** Pratish C Patel, Britany L Raymond, Brandi Cherry, Jennifer Jayaram, Matthew D McEvoy

**Affiliations:** Washington DC VA Medical Center; Vanderbilt University Medical Center; Vanderbilt University Medical Center, Nashville, Tennessee; Vanderbilt University Medical Center, Nashville, Tennessee; Vanderbilt University Medical Center, Nashville, Tennessee; Vanderbilt University Medical Center, Nashville, Tennessee

## Abstract

**Background:**

Enhanced Recovery After Surgery (ERAS) protocols are patient-centered, evidence-based pathways aimed at improving patient outcomes through multidisciplinary interventions in the perioperative period. Among other outcomes, these bundled protocols have been associated with improvement in postoperative immune function, decrease in perioperative inflammatory markers, and a reduction in surgical site infections and hospital readmission.

**Methods:**

ERAS for colorectal surgery was launched at Vanderbilt University Medical Center in 2014. A working group of surgeons, anesthesiologists, infection preventionists, pharmacists, nurses, data analysts, and other staff reviewed the literature to provide evidence and guideline-based recommendations regarding various elements of perioperative care. Following a successful trial period, ERAS principles were sequentially introduced to multiple other surgical services.

**Results:**

To track compliance to protocols and outcome variables associated with ERAS components, we required a method that would allow us to analyze data in real time, as well as customize the reports to align with specific goals of different surgical services. An adaptable data visualization dashboard was developed in Tableau^®^ to meet this need. ERAS components were listed by phase of care and defined by the data source and specific data element. Surgical services could customize their dashboard by selecting data elements to review for informational use only or to include within a scoring system to track performance. An aggregate ERAS protocol view displayed mutual components of at least three service lines to suggest areas for improvement, as well as those of the highest compliance. Monthly and annual compliance trends assist in assessing each metric over time.

ERAS Metric Definitions and Scoring Grid

**Figure:**
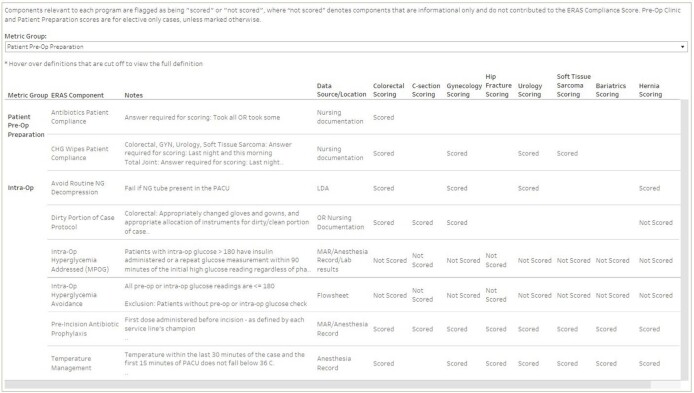

Aggregate ERAS Protocol Opportunities and Wins Summary

**Figure:**
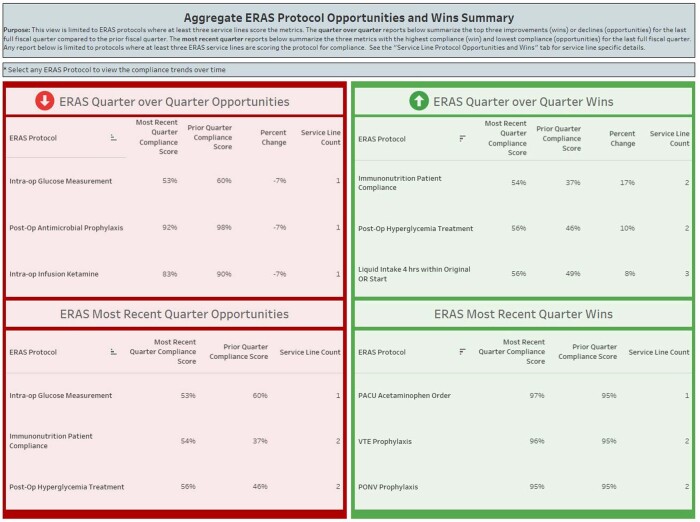

Monthly Compliance by Phase of Care and ERAS Component

**Figure:**
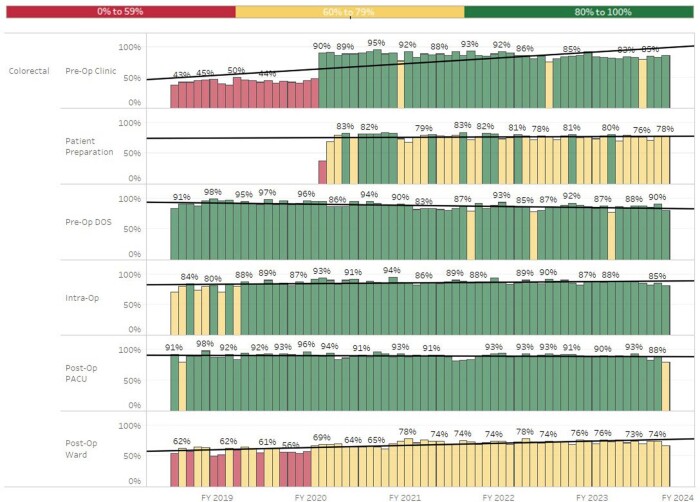

**Conclusion:**

The ERAS component dashboard allowed service line leaders and perioperative administrators to view various key performance indicators such as appropriate perioperative antimicrobial prophylaxis, avoidance of intraoperative hyperglycemia, and preoperative skin disinfection. The data from this dashboard seeks to standardize perioperative care, improve patient recovery, and reduce hospital readmission.

**Disclosures:**

**Pratish C. Patel, PharmD, BCIDP, AAHIVP**, VBI Vaccines: Stocks/Bonds

